# The complete chloroplast genome of *Strobilanthes biocullata* (Acanthaceae)

**DOI:** 10.1080/23802359.2021.1927868

**Published:** 2021-05-19

**Authors:** Qin Wang, Sunan Huang, Xin Chen, Yunfei Deng

**Affiliations:** a College of Biology and the Environment, Co-Innovation Center for Sustainable Forestry in Southern China, Nanjing Forestry University, Nanjing, People’s Republic of China; b Key Laboratory of Plant Resources Conservation & Sustainable Utilization, South China Botanical Garden, Chinese Academy of Sciences, Guangzhou, People’s Republic of China; c University of Chinese Academy of Sciences, Beijing, People’s Republic of China

**Keywords:** *Strobilanthes biocullata*, chloroplast genome, phylogenetic, Acanthaceae

## Abstract

*Strobilanthes biocullata* is a plietesial species endemic to China. The complete chloroplast genome (cp genome) of *S. biocullata* was sequenced for the first time. The cp genome of *S. biocullata* is 144,012 bp in length. It consists of a large single copy (LSC) region (91,628 bp) and a small single copy (SSC) region (17,666 bp), which are separated by two inverted repeats (IRs, 34,718 bp). It contains 114 unique genes, including 80 protein-coding genes, 30 tRNA genes, and four rRNA genes. The overall GC content is 38.2%. Phylogenetic analysis of 13 species has been conducted. This newly sequenced cp genome will be useful to further genetic diversity, phylogeny, and genomic studies of the genus *Strobilanthes*.

*Strobilanthes* Blume (Acanthaceae) is a genus containing about 450 species distributed in the tropical and subtropical regions of Asia with some species extending to Pacific Islands (Hu et al. 2011). *Strobilanthes biocullata* Y. F. Deng & J. R. I. Wood differs from all other species by its bracts having two swollen bulges on the dorsal surface and its plietesial life history which flowered gregariously after growing eight years and then died (Deng et al. 2010). In this paper, the complete chloroplast genome (cp genome) of *S. biocullata* was reported for the first time. The cumulative data have and will continue to contribute to our understanding of the cp genome feature of *Strobilanthes*.

The silica-gel dried leaves of *S. biocullata* were collected from Wudaoshui Zhen, Sangzhi Xian, Hunan Province, China (29°42′18.48″N, 109°55′28.03″E, 778 m). The voucher specimen (Yunfei Deng 27197) is deposited at the herbarium of South China Botanical Garden Herbarium, Chinese Academy of Sciences, Guangzhou, China. Total genomic DNA was extracted from leaves using modified CTAB method (Doyle and Doyle 1987). Short-insert (300–500 bp) libraries were constructed using the Nextera XT DNA Library Prep Kit (Illumina, San Diego, CA) following the manufacturer's instructions. Pair-end (PE) sequencing was performed on the Illumina HiSeq 2500 instruments. To get plastid-like reads, the sequenced clean PE reads were filtered using GetOrganelle pipeline (Jin et al. 2018). The filtered reads were assembled using SPAdes v. 3.11.1 (Bankevich et al. 2012). The genome was automatically annotated using Plastid Genome Annotator (PGA) (Qu et al. 2019), coupled with manual correction in Geneious prime v.2020.0.5 (Kearse et al. 2012). *Andrographis paniculata* (GenBank accession number NC_022451) served as reference for assembly and annotation. The final complete cp genome was submitted to GenBank (accession number MW044601.1).

The complete cp genome of *S. biocullata* is 144,012 bp in length and presents a typical quadripartite structure including a large single copy (LSC) region (91,628 bp), a small single copy (SSC) region (17,666 bp), and two inverted repeat regions (IRs, 34,718 bp). The overall GC content of *S. biocullata* plastome is 38.2%. This plastome contains 114 unique genes, including 80 protein-coding genes, 30 tRNA genes, and four rRNA genes. The *ycf15* gene was considered as pseudogene due to the presence of internal stop codon.

To reconstruct the phylogeny of Acanthaceae, 13 Acanthaceae cp genomes were included and plastomes of two relative families were used as outgroups (Figure 1). The sequences were aligned using MAFFT v. 7.450 (Katoh and Standley 2013). Maximum-likelihood (ML) tree was reconstructed using RAxML v. 8.2.11 (Stamatakis 2014) with the GTR + G+I nucleotide substitution model and all branch nodes were calculated under 1000 bootstrap replicates.

**Figure 1.**
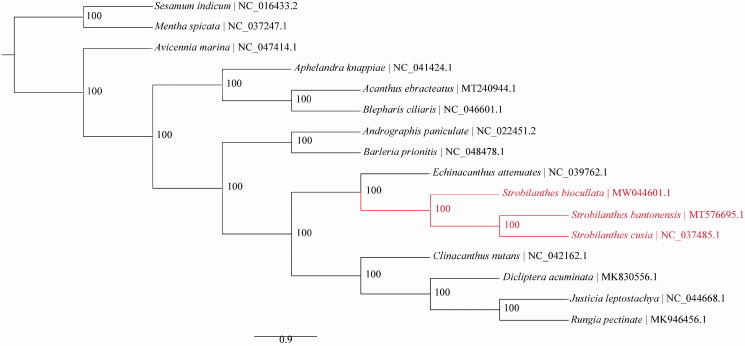
Maximum-likelihood (ML) phylogeny of Acanthaceae based on complete chloroplast genome sequences. Numbers at the right of nodes are bootstrap support values. GenBank accession numbers: *Dicliptera acuminata* MK830556.1, *Rungia pectinate* MK946456.1, *Acanthus ebracteatus* MT240944.1, *Strobilanthes bantonensis* MT576695.1, *Sesamum indicum* NC_016433.2, *Andrographis paniculata* NC_022451.2, *Mentha spicata* NC_037247.1, *Strobilanthes cusia* NC_037485.1, *Echinacanthus attenuatus* NC_039762.1, *Aphelandra knappiae* NC_041424.1, *Clinacanthus nutans* NC_042162.1, *Justicia leptostachya* NC_044668.1, *Blepharis ciliaris* NC_046601.1, *Avicennia marina* NC_047414.1, *Barleria prionitis* NC_048478.1, and *Strobilanthes biocullata* MW044601.1.

The results of the phylogenetics analysis confirmed the monophyly of Acanthaceae as previous studies (McDade et al. 2008) (Figure 1). As expected, *S. bantonensis* and *S. cusia* were more close to *S. biocullata*. All the nodes received 100% bootstrap support. The genome data in this paper can be subsequently used for further genetic diversity, phylogeny, and genomic studies of the genus *Strobilanthes* and will contribute to further phylogeny studies of Acanthaceae.

## Data Availability

The data that newly obtained at this study are openly available in the NCBI (https://www.ncbi.nlm.nih.gov/) under accession number of MW044601.1.
